# Optical Properties of Carbon Dots Synthesized by the Hydrothermal Method

**DOI:** 10.3390/ma16114018

**Published:** 2023-05-27

**Authors:** Marfa Egorova, Aleksandra Tomskaya, Svetlana Afanasyevna Smagulova

**Affiliations:** 1 Institute of Physics and Technologies, North-Eastern Federal University, 677000 Yakutsk, Russia; mn.egorova@s-vfu.ru (M.E.); ae.tomskaya@s-vfu.ru (A.T.); 2 Laboratory of Spectroscopy of Nanomaterials, A.M. Prokhorov General Physics Institute, RAS, 119991 Moscow, Russia; 3 Phystech School of Electronics, Photonics and Molecular Physics, Moscow Institute of Physics and Technology, 141701 Dolgoprudny, Russia

**Keywords:** carbon dots, hydrothermal synthesis, photoluminescence, citric acid, glucose, soot

## Abstract

In this study, the optical and structural properties of carbon dots (CDs) synthesized using a hydrothermal method were investigated. CDs were prepared from various precursors such as citric acid (CA), glucose, and birch bark soot. The SEM and AFM results show that the CDs are disc-shaped nanoparticles with dimensions of ~7 nm × 2 nm for CDs from CA, ~11 nm × 4 nm for CDs from glucose, and ~16 nm × 6 nm for CDs from soot. The TEM images of CDs from CA showed stripes with a distance of 0.34 nm between them. We assumed that the CDs synthesized from CA and glucose consisted of graphene nanoplates located perpendicular to the disc plane. The synthesized CDs contain oxygen (hydroxyl, carboxyl, carbonyl) and nitrogen (amino, nitro) functional groups. CDs have strong absorption in the ultraviolet region in the range of 200–300 nm. All CDs synthesized from different precursors displayed bright luminescence in the blue-green region of the spectrum (420–565 nm). We found that the luminescence of CDs depended on the synthesis time and type of precursors. The results show that the radiative transitions of electrons occur from two levels with energies ~3.0 eV and ~2.6 eV, which are due to the presence of functional groups.

## 1. Introduction

The development of new carbon nanomaterials with luminescent properties is a promising and relevant area of scientific research [1,2,3,4]. Among luminescent carbon nanomaterials (graphene oxide and carbon nanotubes), carbon dots (CDs) have attracted great interest because of their bright, tunable luminescence, high photostability, good biocompatibility, and low toxicity [5]. Owing to these properties, CDs can replace conventional quantum dots [5,6,7] and can be used in many fields of optoelectronics and biomedicine [2,5,8,9,10,11,12]. According to many authors [13,14,15], CDs are quasi-spherical nanoparticles with an average size of less than 10 nm and contain oxygen and nitrogen functional groups. CDs are synthesized from various carbon precursors such as graphite, graphene oxide, carbon nanotubes, citric acid, and even leaves, hair, grass, etc. [16]. CDs are synthesized by various methods such as chemical oxidation, hydrothermal or solvothermal synthesis, pyrolysis, and microwave synthesis. One of the most effective and simple synthesis methods is hydrothermal synthesis because it does not require the use of expensive equipment and materials and it scales well [17]. The hydrothermal method makes it easier to control the conditions for tuning the optical properties of CDs and to vary the luminescence and absorption spectra, chemical composition, and types of functional groups attached to the CDs [18]. In addition, this method is environmentally friendly, which allows it to be used to develop the “green” synthesis CDs in which natural and renewable materials, non-toxic chemicals, and solvents are used as carbon precursors [19]. This method has attracted much attention from researchers, since the introduction of “green” technologies is essential for protecting the environment. Therefore, the improvement of the existing methods for the synthesis of CDs is an urgent task.

The question of mechanisms of luminescence is the subject of discussion by many researchers, for which there is still no unambiguous answer. Some authors attribute the appearance of luminescence to the structure of CDs and others to the luminescence of molecules that are attached to the surface and edges of CDs [20,21,22,23,24]. Additionally, there is no universally accepted model for the structure of CDs. At present, most authors believe that CDs have an amorphous or highly crystalline carbon structure and a shell consisting of oxygen- and nitrogen-containing groups [16,25,26,27,28,29,30].

In this work, we studied the optical and structural properties of CDs synthesized by a hydrothermal method using citric acid (CA), glucose, and birch bark soot. It is shown that CDs are disc-shaped nanoparticles. It was found that the structure and optical properties of the CDs depended on the synthesis time and type of precursors. The luminescence of the synthesized CDs was observed in the blue-green region of the spectrum and depended on the excitation wavelength. The functional groups attached to the carbon dots introduced a number of discrete levels into the energy gap of CDs. It is assumed that radiative transitions of electrons occur with the participation of discrete levels associated with functional groups.

## 2. Materials and Methods

Carbon dots were synthesized by a hydrothermal method from citric acid, glucose, and soot of birch bark. The same ratios of reagents were used for the synthesis of all CDs: 1 g of carbon precursor, 6 mL of an aqueous solution of ammonia NH_4_OH (25%), and 15 mL of deionized water (DI). The resulting solutions were transferred into a 50 mL Teflon-lined stainless autoclave. The autoclave was placed in a universal oven and heated at 180 °C for different durations. After the reaction, the autoclave was allowed to cool to room temperature. The final solutions were dialyzed in a dialysis bag (3.5 kDa, MWCO) for approximately 12 h in DI water at room temperature to remove excess synthesis products. After dialysis, the solution was filtered through a track membrane with a pore size of 100 nm. The color of the solutions of CDs obtained from glucose and CA was brown, and the CDs from soot were transparent with a yellowish tint.

The surface morphology and size of the CDs were studied by atomic force microscopy (AFM) on an Ntegra spectra spectrometer (NT-MDT, Zelenograd, Moscow, Russia). The CDs’ structures and lateral dimensions were studied using a JEOL JSM 7800-F scanning electron microscope (SEM) (JEOL, Tokyo, Japan) and JEOL-2010 transmission electron microscope (TEM) (JEOL, Tokyo, Japan). To study the composition of the functional groups of the CDs, infrared spectra (IR) were measured on an FTS 7000 IR-Fourier spectrometer (Varian, Palo Alto, CA, USA) and on a Spotlight 200i FTIR Microscopy System spectrometer (Perkin Elmer, Waltham, MA, USA) in the wavelength range of 4200–400 cm^−1^. X-ray photoelectron spectroscopy (XPS) was used to study the quantitative composition of the functional groups. The measurements were performed on a SPECS photoelectron spectrometer (SPECS GmbH, Berlin, Germany), using a PHOIBOS-150-MCD-9 hemispherical analyzer (MgK* radiation, h* = 1253.6 eV, 150 W). The binding energy scale was pre-calibrated to the positions of the Au_4_f7/2 (84.00 eV) and Cu_2_p3/2 (932.67 eV) island-level peaks. Measurements of the Raman scattering spectra were carried out on an Ntegra spectra setup (NT-MDT, Zelenograd, Moscow, Russia) at an excitation laser wavelength of 532 nm. Luminescence spectra were measured using a Perkin Elmer LS 50 B spectrometer (Perkin Elmer, Waltham, MA, USA). UV-VIS absorption spectra were measured on a Lambda 750 spectrophotometer (Perkin Elmer, Waltham, MA, USA).

## 3. Results and Discussion

**Figure 1:**
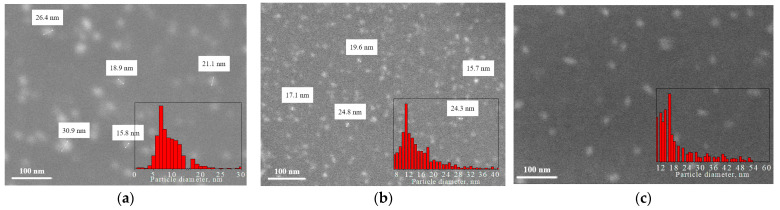
SEM images and size distributions of (**a**) CDs from CA; (**b**) CDs from glucose; (**c**) CDs from soot.

The structures of the CDs were studied using SEM. To perform this, CDs from the solution were deposited on silicon substrates and dried in air atmosphere. Figure 1 shows SEM images of CDs synthesized from CA, glucose, and birch bark soot and their size distributions (insets). It was shown that the forms of the CDs synthesized from different precursors were different. Carbon dots from CA and glucose are round and well dispersed, with an average size of ~7 nm (CDs from citric acid) and ~11 nm (CDs from glucose). During the synthesis of CDs from glucose and CA, CDs are formed by the “bottom-up” method from carbon atoms in a highly carbonized aqueous solution. In solution, the process of self-assembly of carbon atoms occurs, which leads to the appearance of graphene sp^2^ planes. CDs from soot have round (~16 nm) or elongated (~16 × 6 nm) shapes. This may be because the CDs from soot are located differently on the surface of the SiO_2_/Si substrate. In contrast to the CDs from CA and glucose, CDs from soot are obtained by splitting large fragments of soot. Perhaps in this case, the CDs’ cores had graphite structures. It was found that the type of precursor affects the size and shape of the CDs, and there is also an influence of the CD assembly method.

**Figure 2:**
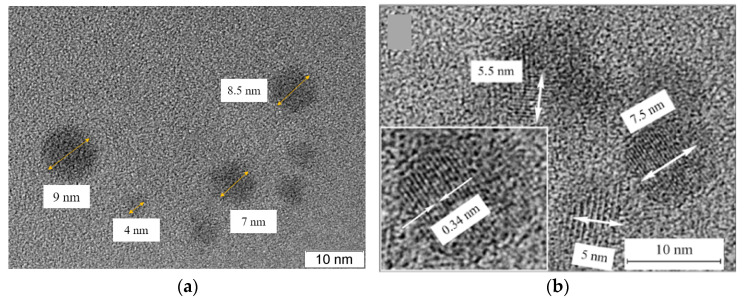
(**a**) TEM and (**b**) HR-TEM images of CDs from CA.

Figure 2 shows the TEM images of the CDs from CA. As shown, the shapes of the obtained CDs are round, with an average diameter of ~7 nm (Figure 2a). High-resolution TEM images (Figure 2b) illustrate ~15 stripes on the CDs, and the distance between them is observed at 0.34 nm. Despite the fact that the distance between graphene nanoplates is 0.34 nm, in our opinion CDs do not have a graphite structure since it is known [31] that graphite lattice strongly quenches luminescence. Such stripes in CDs are also observed in other studies, in which the distance between the strips varies in the range from 0.21 nm to 0.34 nm [5,11,25,31,32].

**Figure 3:**
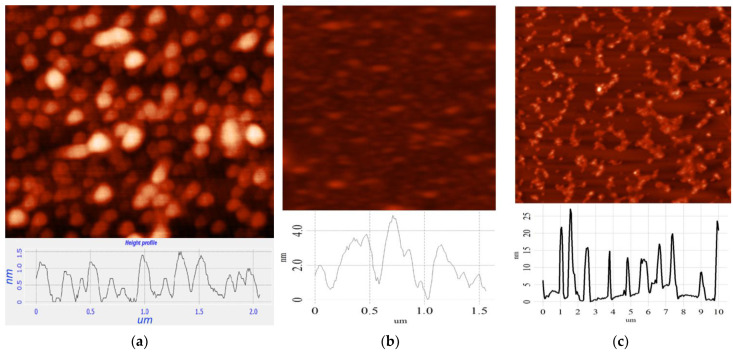
AFM of CDs (**a**) from CA; (**b**) from glucose; (**c**) from soot.

The thicknesses of the CDs were measured using AFM. As shown in Figure 3, the average thickness of CDs from CA is ~2 nm (Figure 3a) and that from glucose is ~4 nm (Figure 3b). For CDs from soot, the thickness varied from 6 to 20 nm (Figure 3c).

A comparison of the obtained experimental data from SEM, TEM, and AFM allowed us to assume that the CDs were disc-shaped nanoparticles. The stripes visible in the TEM image (Figure 2b) are probably associated with the edges of the graphene nanoplates coated perpendicular to the disc plane, with a distance of 0.34 nm between them. The size of one graphene nanoplate in the case of CDs from CA was ~7 nm × 2 nm, and in the case of CDs from glucose, it was ~11 nm × 4 nm.

**Figure 4:**
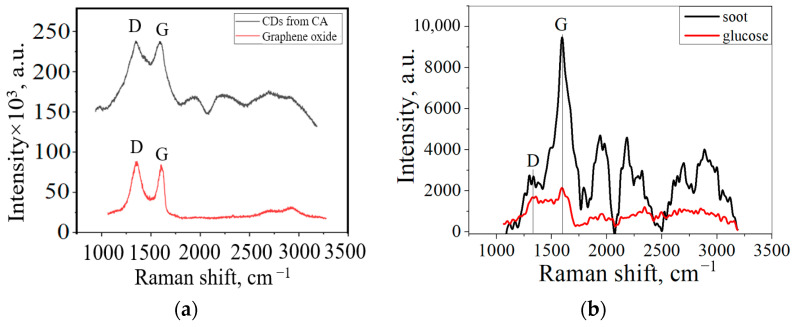
Raman spectra of (**a**) CDs from CA and GO; (**b**) CDs from soot and glucose.

The graphene nanoplates on the CDs were mainly oxidized along the edges, adding a number of functional groups. Figure 4a shows the Raman spectra of the CDs obtained from CA and graphene oxide (GO). The Raman spectra of the CDs showed that the CDs’ structures were similar to those of GO. They exhibit two peaks at 1348 cm^−1^ and 1588 cm^−1^, which correspond to the D and G bands of carbon materials, respectively. Figure 4b shows the Raman spectra of carbon obtained from soot and glucose. It is shown that in the CDs from soot, the intensity of the G peak is 3.5 times greater than that of the D peak, which indicates its graphite structure.

**Figure 5:**
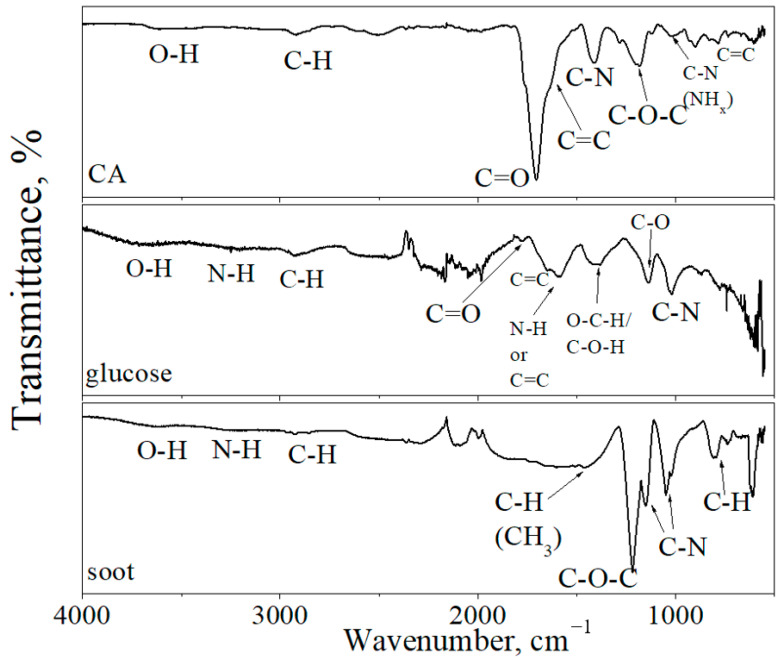
FTIR spectra of CDs from CA, glucose, and soot.

Figure 5 shows the IR spectrum of CDs. In the IR spectra of all CDs, the absorption peaks observed at 3500–3700 cm^−1^ and 2840–3000 cm^−1^ are assigned to O–H and C–H stretching vibrations of carboxylic acid and amine groups, respectively. Absorption bands at 1085–1225 cm^−1^ are assigned to C–O–C stretching vibrations. The peaks in this region can also be assigned to the C-NH-C asymmetric stretching vibrations [33]. The IR spectrum of CDs from CA demonstrates the C=C stretching vibrations in isolated sp^2^ carbon domains at 1680–1610 cm^−1^ [34]. The broad absorption bands at 1400–1423 cm^−1^ are assigned to C–N stretching vibrations, and the narrow absorption band at 1553–1565 cm^−1^ is assigned to N–H bending vibrations [35,36]. In the IR spectra of CDs from CA, an intense peak at 1703 cm^−1^ was assigned to C=O stretching vibrations [35,37], which were not detected in CDs from soot, and in CDs from glucose, the intensity of this peak was very low.

In the IR spectra of CDs from soot, the absorption peaks at 1465 cm^−1^, 1217 cm^−1^, and 1250–1020 cm^−1^ are assigned to C–H, C–O–C, and C–N (amine) vibrations, respectively. Weak stretching vibrations of the N–H bond were also observed, which are additional to the background of C–H stretching vibrations. The IR spectra of the carbon dots from glucose contain peaks at 1588 cm^−1^ and 1386 cm^−1^, which indicate N–H and C–O–H bonds [38,39]. As shown by the IR spectra (Figure 5) of the CDs from CA, the main functional group was the carbonyl group (C=O). In the CDs from soot, the dominant group was the ether group (C–O–C).

**Figure 6:**
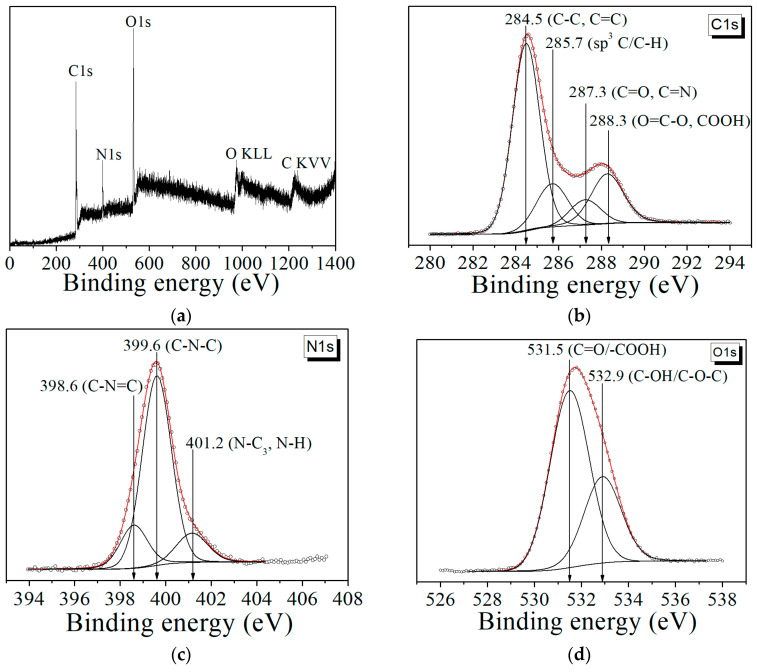
XPS spectra of CDs from CA: (**a**) overview XPS profile; (**b**) C1s spectrum; (**c**) N1s spectrum; (**d**) O1s spectrum.

XPS was employed to characterize the surface groups of the CDs to investigate their composition. Figure 6a shows an overview of the photoelectronic spectrum of the CDs, which are composed of carbon (C1s), nitrogen (N1s), and oxygen (O1s and O KLL). Figure 6b shows the C1s XPS spectra of the CDs obtained from CA. The C1s spectra can be fitted of four kinds of C species: sp^3^ C/C–H (285.8–285.1 eV), C–C/C=C (284.5 eV), C=O and COOH (288.2–288.4 eV), and C=O, C=N (287.2–287.4 eV) [40].

The N1s spectra (Figure 6 c) mainly consist of three peaks: pyridine nitrogen (C–N=C) (398.1–399.3 eV), pyrrole nitrogen (C–N–C) (399.5–400.2 eV), and an amide group (graphitic nitrogen) N–C_3_/N–H (401.7–402 eV). The O1s spectrum peaks at 531.5 eV and 532.9 eV could be assigned to C=O/–COOH and C–OH/C–O–C [19,40] (Figure 6d). XPS analysis confirmed the data obtained with IR in the presence of oxygen and nitrogen-containing functional groups in the CDs.

**Figure 7:**
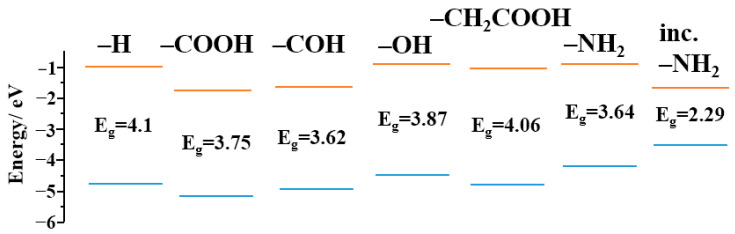
Energy levels of CDs with functionalization by oxide and amino groups.

It is known [41] that functional groups introduce a number of discrete levels into the energy gap of CDs. The levels associated with the presence of functional groups were calculated. The calculation was carried out according to density functional theory (Becke, 3-parameter, Lee-Yang-Parr hybrid functional, using the VWN1 correlation—Vosko, Wilk, and Nusair) [42]. Figure 7 shows that the functional groups introduced a number of local levels into the CDs’ energy system, which possibly participated in the radiative transitions of electrons that caused luminescence.

**Figure 8:**
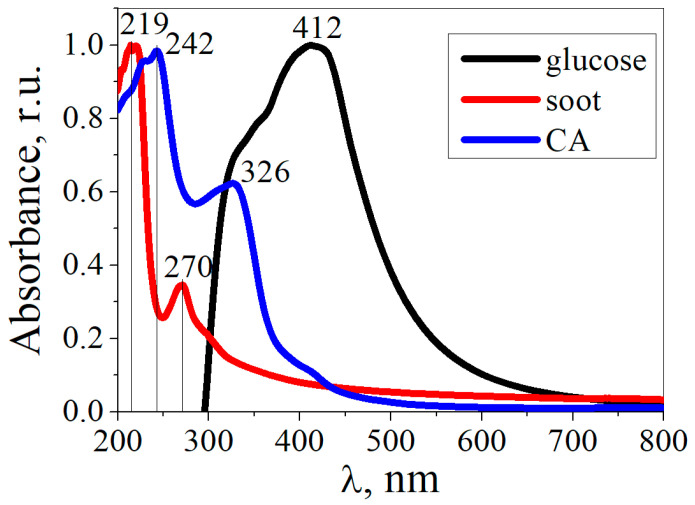
UV-VIS absorption spectra of CDs.

Figure 8 shows the absorption spectra of the CDs obtained from CA, glucose, and soot. The absorption spectra of the CDs from CA and soot differed from those of the CDs from glucose. The CDs from CA exhibited absorption bands at 242 nm and 326 nm, which were assigned to the π→π* transition of sp^2^ aromatic domains and to the n→π* transition of the C=O bond, respectively [43,44]. The absorption band at 412 nm in the CDs from the glucose can be attributed to the n→π* transitions of functional groups on the surface of the CDs. For CDs from soot, the absorption bands in the region from 200 to 290 nm are assigned to π→π* transitions in the C=C bonds of the sp^2^ plane [45].

**Figure 9:**
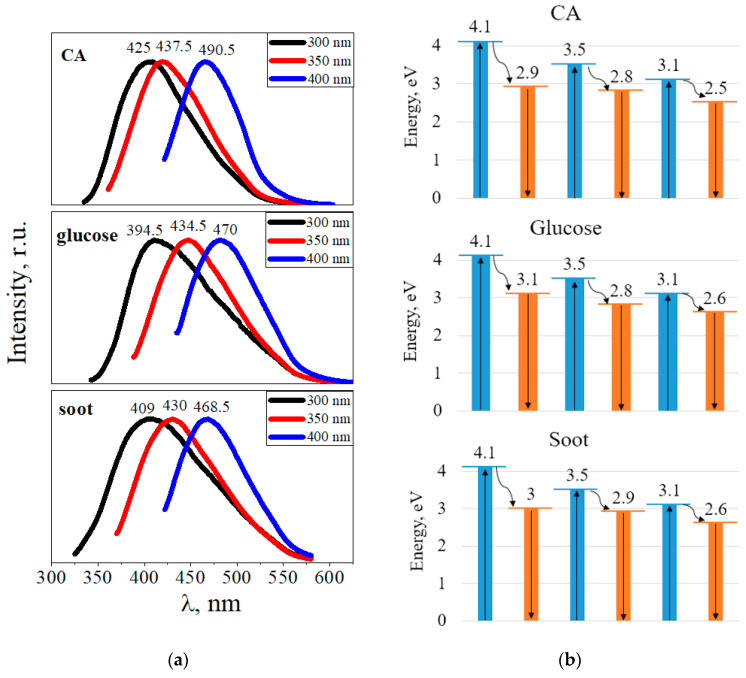
(**a**) Luminescence spectra of CDs from citric acid, glucose and soot; (**b**) transitions of excited electrons to the ground state (blue indicates the transition of electrons upon absorption of a photon, orange indicates radiative transitions).

All CDs synthesized using the hydrothermal method exhibited bright luminescence in the blue-green region of the spectrum. Figure 9a shows the luminescence spectra of the CDs from CA, glucose, and soot depending on the excitation wavelength. When the excitation wavelength changes from 300 to 400 nm, the luminescence peaks shift to the long-wavelength region of the spectrum. For example, upon excitation at a wavelength of 300 nm, CDs from CA luminesce at 425 nm (2.9 eV), glucose at 394.5 nm (3.1 eV), and soot at 409 nm (3.0 eV). Moreover, when excited at a wavelength of 400 nm, CDs from CA luminesce at 490.5 nm (2.5 eV), CDs from glucose at 470 nm (2.6 eV), and CDs from soot at 468.5 nm (2.5 eV).

Figure 9b shows the energies of the radiative transitions of the electrons. After excitation at a wavelength of 300 nm (4.1 eV), radiative transitions of electrons are observed with an energy of 2.9 eV for CDs from CA, with an energy of 3.1 eV for CDs from glucose, and 3 eV for CDs from soot. Upon excitation at a wavelength of 350 nm (3.5 eV), electron transitions occur with energies of 2.8 eV, 2.8 eV, and 2.9 eV for CDs from CA, glucose, and soot, respectively. Upon excitation at a wavelength of 400 nm (3.1 eV), electrons pass with energies of 2.5 eV for CDs from CA, 2.6 eV for CDs from glucose, and 2.6 eV for CDs from soot. We assumed that there are two levels with energies of ~3.0 eV and ~2.6 eV, from which radiative transitions of electrons to the ground state occur. First, from the excited state, the electrons non-radiatively pass to these levels, followed by a radiative transition, which causes luminescence in the CDs. We found that the luminescence peaks did not depend on the type of the precursor. The resulting luminescence spectra were rather broad, with a full width at half maximum of ~100 nm. Changes in the position of the luminescence peak at different excitation wavelengths for CDs are possibly associated with a different set of functional groups or with the summation of several luminescence peaks with similar energies.

**Figure 10:**
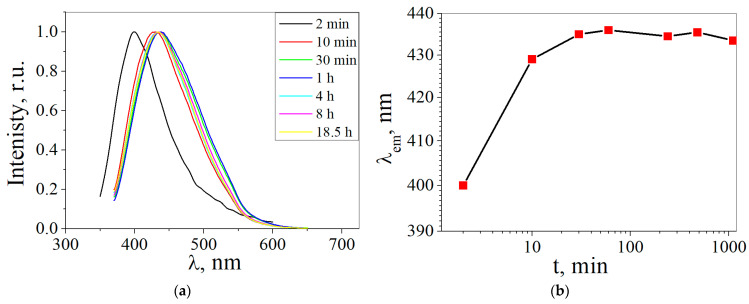
(**a**) Luminescence spectra of CDs from glucose at the different synthesis times (λ_ex_ = 350 nm); (**b**) the dependence of the luminescence of CDs from glucose on the time of synthesis (Red squares indicate the value of the maximum luminescence peak).

The influence of the synthesis time on the luminescence peaks of the CDs synthesized from glucose was determined. Figure 10a shows the luminescence spectra of the CDs obtained from glucose at different synthesis times. It is shown that CDs’ luminescence is at a wavelength of approximately 400 nm at synthesis times from 2 min, while at times from 10 min up to 18.5 h, the CDs’ luminescence is in the region of ~430–435 nm. It has been suggested that during synthesis with a duration of 2 min in a CD solution, one type of luminescence center is formed with a peak at approximately 400 nm, while during synthesis with a duration of 30 min to 18.5 h it is transformed into the second type of luminescence center.

## 4. Conclusions

The hydrothermal method was used to synthesize CDs from CA, glucose, and birch bark soot. The size and shape of the CDs depended on the precursors. CDs are disc-shaped nanoparticles consisting of graphene nanoplates located perpendicular to the disc plane, with distances of 0.34 nm between them. CDs contain oxygen (hydroxyl, carboxyl, carbonyl) and nitrogen (amino, nitro) functional groups. CDs from CA and soot exhibit strong absorption in the UV region. All CDs synthesized from different precursors exhibited bright luminescence in the blue-green region of the spectrum (from 420 nm to 565 nm). An assumption is made that the luminescence of CDs is due to radiative transitions of electrons from two levels with energies of ~3.0 eV and ~2.6 eV. CDs were synthesized from glucose luminescence with a wavelength of approximately 400 nm at synthesis times from 2 min, while at times from 30 min to 18.5 h, the luminescence peak was located in the region of 435 nm.

## Data Availability

The data presented in this study are available upon request from the corresponding author. The data are not publicly available due to the data protection policy of the university.
